# No Weekday Effect in Bariatric Surgery—a Retrospective Cohort Study

**DOI:** 10.1007/s11695-022-06041-9

**Published:** 2022-04-05

**Authors:** Martin L. Skogar, Erik Stenberg, Magnus Sundbom

**Affiliations:** 1 Department of Surgical Sciences, Uppsala University, 751 85 Uppsala, Sweden; 2 Department of Surgery, Faculty of Medicine and Health, Örebro University, Örebro, Sweden

**Keywords:** Weekday, Bariatric surgery, Roux-en-Y gastric bypass (RYGB), Sleeve gastrectomy (SG)

## Abstract

**Purpose:**

Major abdominal surgery carried out in the later part of the week has been associated with increased complication rates. The aim of this study was to explore whether the weekday of surgery affects the 30-day complication risks after primary Roux-en-Y gastric bypass (RYGB) and sleeve gastrectomy (SG).

**Material and Methods:**

Prospectively collected data, extracted from the Scandinavian Obesity Surgery Registry (SOReg), of all patients who underwent primary laparoscopic RYGB or SG between 2010 and 2017 were included in this retrospective cohort study. Multivariate logistic regression adjusted for differences in case-mix and operating center by weekday of surgery.

**Results:**

In total, 49,349 patients were included in this study. The overall 30-day complication rate was 7.2% (*n* = 3574), whereof 2.9% (*n* = 1428) had a severe complication, i.e., requiring intervention in general anesthesia or more. The 30-day mortality rate and readmission rate were 0.02% (*n* = 12) and 7.6% (*n* = 3726), respectively. The highest overall complication rate was seen in patients operated on Wednesdays and Thursdays (7.7%), while severe complications were most common on Wednesdays (3.3%). However, a large variation in severe complications was seen between centers, from 0.4 to 8.0%. After adjustment for case-mix and operating center, there was no significant increased risk of overall complications, severe complications, or readmission rates by weekday of surgery, except for a lower readmission rate in patients operated on Tuesdays.

**Conclusion:**

The result of the present study supports the notion that bariatric surgery can be performed safely on all weekdays.

**Graphical Abstract:**

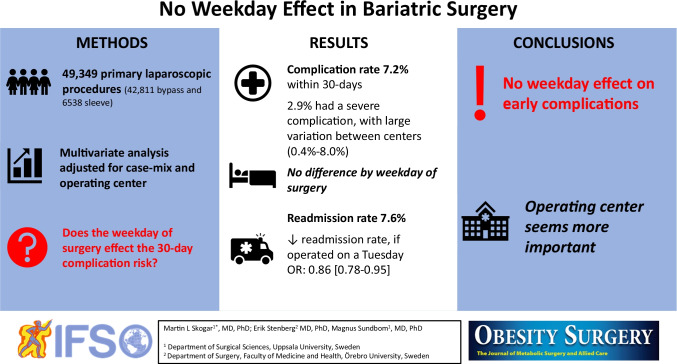

## Introduction

All individuals undergoing surgery fear suffering a complication. Which patient-related factors that affect the complication risk for a specific procedure is often well-known [1–3]. The influence of external factors such as the weekday of surgery on postoperative outcome is less studied. Surgery for gastrointestinal malignancies carried out in the later part of the week (Wednesday–Friday) has been associated with increased complication rates, e.g., gastrectomy for gastric cancer [4], and inferior long-term survival in esophageal [5], hepatobiliary, colorectal [6], and ovarian cancer [7]. In thoracic surgery, coronary artery bypass grafting (CABG) on Fridays has been associated with higher 30-day mortality when compared to Mondays [8]. Likewise, a systematic review and a retrospective study of various elective surgical procedures at English public hospitals found a higher risk of postoperative mortality for patients operated on Fridays and during weekends [9, 10]. Whether the weekday of surgery has any influence on risk of complications after bariatric surgery, a rather technically demanding procedure performed in increasing numbers worldwide, has not yet been studied.

Roux-en-Y gastric bypass (RYGB) and sleeve gastrectomy (SG) are the most commonly performed bariatric procedures [11], both resulting in long-term weight reduction and resolution of metabolic comorbidities for many patient [12, 13]. The risk of severe postoperative complications after bariatric surgery is low, about 3% in Sweden [14]. We have recently reported that having RYGB surgery during the start-up period after summer vacation is associated with an increased risk of serious postoperative complications (Fall et al., in manuscript). In line with this, the complication risk could perhaps be elevated for patients having surgery in the very beginning of the working week, also known as a “Monday product” in Sweden. This expression is similar to the American “Friday afternoon model,” implying that a non-functioning device was assembled on a Friday afternoon when the fitter was tired and dreaming of the weekend, or in the Swedish case, tired after having a busy weekend. Thus, a weekday effect is a general concern.

The aim of this study was to explore whether the weekday of surgery affects the 30-day complication risk after primary laparoscopic RYGB and SG, two technically demanding high-volume procedures performed worldwide in increasing numbers.

## Methods

### Study Design

This retrospective cohort study was based on prospectively collected data extracted from the Scandinavian Obesity Surgery Register (SOReg). All Swedish centers for bariatric surgery report to the registry and individual data for patients undergoing bariatric surgery are continuously collected as part of clinical practice. The database captures baseline data (e.g., age, gender, obesity-related comorbidities, date of surgery, and type of procedure) and outcome at subsequent follow-up at 6 weeks and 1, 2, 5, and 10 years. Since the start in 2007, SOReg has included 97.4% all bariatric procedures performed in Sweden with an internal validity of 99% [15].

All patients who underwent primary laparoscopic RYGB or SG surgery between 2010 and 2017 were included. Patients operated on during weekends (*n* = 48) were excluded because elective surgery during weekends is not common practice in Sweden and may represent a specific selection of patients.

### Outcomes

The primary outcome was severe complications within 30 days of surgery, defined as complications graded as Clavien-Dindo IIIb or worse, i.e., a complication requiring intervention under general anesthesia, resulting in organ failure, or death [16]. Secondary outcomes were 30-day overall complication and readmission rates. Readmission was defined as hospitalization (any cause) within 30 days after surgery. Analysis of specified postoperative complications (leak, abscess, bleeding, bowel obstruction, cardiovascular event, DVT, pulmonary complication) by weekday was also performed.

### Statistics

Chi^2^ test was used for univariate analyses of categorical variables. Univariable and multivariable logistic regression was used to examine the unadjusted and adjusted odds ratios (OR) and 95% confidence intervals (CIs) by weekday, using Monday as the reference category. The multivariable model adjusted for differences in operating center, type of procedure, gender, age, body mass index (BMI), year of surgery, smoking status and obesity-related comorbidities such as diabetes, hypertension, depression and acid-related conditions, (dichotomized on ongoing medication or not), and sleep apnea (use of continuous positive airway pressure, CPAP) as well as history of deep vein thrombosis (DVT). Missing values were handled by listwise deletion. All *p* values were 2-sided and *p* value < 0.05 was considered statistically significant. All analyses were performed using IBM SPSS Statistics version 28.

## Results

In total, 49,349 patients having had their first bariatric procedure were included in the analysis, whereof 42,811 had RYGB and 6538 SG. The operations were performed at 52 different centers (20 to 6517 procedures per center) and the 30-day severe complication rate varied between 0.4 and 8.0% per center.

The overall 30-day complication rate was 7.2% (*n* = 3574), whereof 2.9% (*n* = 1428) had a severe complication. The 30-day mortality rate and readmission rate were 0.02% (*n* = 12) and 7.6% (*n* = 3726), respectively.

Most procedures were performed in the beginning of the week with a falling trend throughout the week. Significant differences in patient characteristics were found by day of surgery for type of procedure, gender distribution, age, BMI, year of surgery, smoking status, diabetes, hypertension, and acid-related conditions (Table 1).

**Table 1:** Characteristics by weekday of the 49,349 patients included in the study cohort

		Monday (%)	Tuesday (%)	Wednesday (%)	Thursday (%)	Friday (%)	Unadjusted *p* value
Total number of procedures		15,100	14,055	12,033	7148	1013	
Operation	LRYGB	86.5%	82.7%	90.3%	88.7%	89.3%	< 0.01
	LSG	13.5%	17.3%	9.7%	11.3%	10.7%	
Gender	Female	75.5%	77.0%	76.5%	76.6%	78.0%	0.03
	Male	24.5%	23.0%	23.5%	23.4%	22.0%	
Age (year)	< 30	20.5%	19.9%	21.1%	21.7%	18.6%	< 0.01
	30–40	26.4%	27.4%	27.5%	26.7%	28.6%	
	40–50	30.8%	31.2%	30.8%	29.7%	31.7%	
	50–60	18.8%	18.2%	17.7%	18.3%	18.3%	
	> 60	3.5%	3.3%	2.9%	3.6%	2.9%	
BMI (kg/m^2^)	< 40	40.8%	43.5%	38.7%	42.4%	44.6%	< 0.01
	40–50	50.4%	49.6%	54.2%	51.2%	50.4%	
	50–60	7.8%	6.2%	6.5%	5.8%	4.9%	
	> 60	1.0%	0.7%	0.6%	0.5%	0.0%	
Year of surgery	2010	13.5%	13.7%	13.9%	12.4%	19.2%	< 0.01
	2011	15.1%	14.8%	17.2%	14.7%	26.0%	
	2012	15.1%	13.4%	15.1%	13.5%	16.7%	
	2013	15.3%	14.3%	15.0%	13.5%	16.3%	
	2014	13.8%	13.1%	12.3%	13.9%	10.8%	
	2015	12.2%	12.2%	11.5%	13.7%	5.6%	
	2016	10.0%	12.1%	9.5%	12.4%	4.5%	
	2017	4.9%	6.3%	5.5%	6.0%	0.9%	
Smoking	Yes	10.4%	11.5%	10.7%	11.9%	11.2%	< 0.01
	Missing data	32.4%	28.1%	32.1%	27.5%	38.6%	
Diabetes*		14.2%	13.0%	13.5%	13.5%	11.0%	< 0.01
Sleep apnea*		10.2%	9.6%	9.9%	9.6%	8.1%	0.14
Hypertension*		25.9%	24.4%	25.2%	25.1%	22.8%	0.02
Depression*		15.5%	15.2%	15.3%	15.5%	15.3%	0.92
Acid-related conditions*		9.6%	10.2%	9.9%	11.3%	7.7%	< 0.01
History of DVT		2.5%	2.5%	2.4%	2.2%	3.1%	0.31

*BMI* body mass index, *DVT* deep vein thrombosis, *LRYGB* laparoscopic Roux-en-Y gastric bypass, *LSG* laparoscopic sleeve gastrectomy

^*^Defined by ongoing medication (use of continuous positive airway pressure, CPAP for sleep apnea)

### Complications and Adverse Events

The highest rate of severe complications was seen in patients operated on Wednesdays (3.3%), while overall complications were most common on Wednesdays and Thursdays (7.7%). Among the studied specific complications, bowel obstruction was more common in patients operated on Wednesdays (unadjusted analyses). After adjustment for case-mix and operating center, there was no significant increased risk of severe complications, overall complications, or readmission rates by weekday of surgery, except for a lower readmission rate in patients operated on Tuesdays (*p* < 0.01) (Table 2).

**Table 2:** Complications and adverse events by weekday

		Monday	Tuesday	Wednesday	Thursday	Friday	Unadjusted *p* value
Total number of procedures		15,100	14,055	12,033	7148	1013	
Severe complications*	*N* (%)	417 (2.8)	368 (2.6)	394 (3.3)	217 (3.0)	32 (3.2)	0.02
	Unadjusted (OR)	Ref	0.95 (0.82–1.09)	1.19 (1.04–1.37)	1.10 (0.93–1.30)	1.15 (0.80–1.66)	
	Adjusted (OR)	Ref	0.95 (0.81–1.11)	1.10 (0.94–1.28)	1.00 (0.83–1.19)	0.92 (0.62–1.36)	
Overall complications	*N* (%)	1085 (7.2)	942 (6.7)	922 (7.7)	551 (7.7)	74 (7.3)	0.02
	Unadjusted (OR)	Ref	0.93 (0.85–1.02)	1.07 (0.98–1.17)	1.08 (0.97–1.20)	1.02 (0.80–1.30)	
	Adjusted (OR)	Ref	0.91 (0.83–1.01)	1.09 (0.98–1.20)	0.97 (0.86–1.08)	0.91 (0.70–1.18)	
Readmission	*N* (%)	1102 (7.3)	1047 (7.4)	923 (7.7)	568 (7.9)	86 (8.5)	0.32
	Unadjusted (OR)	Ref	1.02 (0.94–1.12)	1.06 (0.96–1.15)	1.10 (0.99–1.22)	1.18 (0.94–1.48)	
	Adjusted (OR)	Ref	0.86 (0.78–0.95)	0.95 (0.86–1.05)	0.96 (0.85–1.08)	0.99 (0.77–1.27)	
Specific complications (*N*, %)	Leak	127 (0.8)	113 (0.8)	115 (1.0)	71 (1.0)	10 (1.0)	0.53
	Abscess	102 (0.7)	77 (0.5)	88 (0.7)	57 (0.8)	5 (0.5)	0.19
	Bleeding	257 (1.7)	219 (1.6)	222 (1.8)	128 (1.8)	14 (1.4)	0.38
	Bowel obstruction	145 (1.0)	127 (0.9)	150 (1.2)	56 (0.8)	9 (0.9)	0.01
	Cardiovascular event	21 (0.1)	22 (0.2)	9 (0.1)	10 (0.1)	4 (0.4)	0.06
	DVT/PE	14 (0.1)	11 (0.1)	9 (0.1)	8 (0.1)	0 (0)	0.77
	Pulmonary complication	63 (0.4)	80 (0.6)	67 (0.6)	44 (0.6)	5 (0.5)	0.26

*DVT/PE* deep vein thrombosis/pulmonary embolism

^*^Defined by complications graded as Clavien-Dindo IIIb or worse, i.e., a complication requiring intervention under general anesthesia, treatment at an intensive care unit, or death

## Discussion

In this large national cohort of patients undergoing primary laparoscopic bariatric surgery, an expanding elective high-volume procedure, the highest rate of both severe and overall complications was seen in patients operated in the middle of the week. However, when adjusting for case-mix and operating center, these associations were lost, except for a lower readmission rate in patients operated on Tuesdays.

There are no previous studies of a “weekday effect” in bariatric surgery. As previously mentioned, several studies on malignant surgery have found an association of worse patient-outcome for procedures carried out in the later part of the working week, both regarding short- and long-term outcomes. However, there are several studies contradicting this finding, both for malignancies and other elective surgical procedures. In esophagogastric cancer surgery, three large population-based cohort studies from the Netherlands could not find any association between weekday of surgery and short- or long-term outcomes [17–20], nor in two studies of pulmonary resections [21, 22]. Likewise, in a study of 58,646 elective abdominal surgeries, no association between weekday of surgery and adverse outcome was found [23]. These diverging results are likely multifactorial and depending on which confounders that are adjusted for. Operating center is likely an important confounder as differences in which day of the week hospitals routinely perform major elective abdominal surgery influence complication rate because of the significant hospital variation in outcomes after surgery [24]. In our data, the 30-day severe complication rates varied from 0.4 to 8.0% between hospitals. We therefore believe that adjusting for operating center, as done here, is utterly important.

The present results can also be interpreted as successful patient selection, i.e., scheduling less demanding procedures in the end of the week, when hospital resources and preparedness for complications are diminishing due to the approaching weekend. This notion is supported by the lower BMI and lower prevalence of comorbid diseases such as diabetes and acid-related conditions in patients operated on Fridays compared to Mondays, as well as lower ratio of men, often having increased complication risks due to abdominal obesity. These factors have, together with older age and smoking, been associated with an increased risk of complications after bariatric surgery [1, 25, 26].

The difference in readmission rates could depend on reduced access to surgeons and nurses specialized in obesity care at the end of the week, hence successfully dealing with problems occurring in patients operated on Mondays and Tuesdays, before leaving the rest to emergency staff during weekends. Small initial problems in oral intake, due to the altered gastrointestinal anatomy and physiology after bariatric surgery, can be very difficult to observe during the short postoperative length of stay, often only one night in hospital. To overcome this, many centers call their patients repeatedly during the first week of surgery, while others have early obligatory out-patient visits.

### Strength and Limitations

The large sample size from a nation-wide cohort of primary laparoscopic RYGB and SG with high completeness of follow-up is among the strengths of the present study. The multivariable logistic regression model adjusting for differences in case-mix and operating center is another major strength, especially since our unadjusted data demonstrated an increased complication risk for patients operated in the later part of the week. However, as in any registry-based study, there is an inherent risk of residual confounding factors not adjusted for. Not being able to assess individual surgeons, nor their experience, limits the analysis. Previous studies have demonstrated that less experienced first assistants increases the 30-day readmission rate and the need for intensive care [27]. Our result is strengthened by the retrospective description of everyday surgery on a national basis, thus not possible to manipulate by including only selected patients in a prospective study. Other strengths are the use of very similar surgical techniques at all centers, for example, the double omega-loop technique for RYGB (50-cm biliopancreatic limb and a 100-cm alimentary limb) [28] and the well-defined definitions of complications in the national SOReg registry, which in turn has a high acquisition rate and validity. Finally, reporting negative results may raise questions on study power. However, the study had adequate power to reveal clinically relevant risk differences (> 1%) in overall complications for all weekdays except for Friday.

## Conclusion

This large registry-based study found no association between weekday of surgery and the risk of complication in patients having first-time laparoscopic bariatric surgery, when adjusting for case-mix and operating center. Although the results support the notion that bariatric surgery can be performed safely on all weekdays, a large variation in severe complication rate was seen between centers, from 0.4 to 8.0%.
